# Valyl

**Published:** 1902-05

**Authors:** 


### ABSTRACT.

### Valyl.

(VALERIANIC ACID DIETHYLAMID.)

The many uses to which valerian and its preparations can be put have been offset by the uncertainty of its action, together with the disagreeable taste, odor and the eructations, thus limiting its use.

In the treatment of hysteria and a train of nervous disorders it has been found extremely valuable where other remedies have failed. The necessity for a uniform product in a concentrated form, which could be given without gastric and other disturbances to the patient, has been conceded, but until now, not accomplished.

In valyl, or chemically, the diethylamid of valerianic acid, this result has been obtained. Experience with valyl at the medical clinic of the University of Breslau, (Geheimrath Kast) showed that it had all the virtues of valerian and valerianic acid in the treatment of hysteria and other nervous disorders, as well as of the vasomotor system; while Prof. Pfannenstiel in the medical ward of the central prison (privat docent Bonhoffer) Breslau, as well as in private practice, found it useful in: (1) cases of hysteria and functional heart troubles; (2) cases of traumatic neuroses; (3) cases of migrain, hemicrania, neuralgia and ischuria; (4) in cases of troublesome menstruation, where abdominal pains and headaches were relieved.

Valyl is offered in gelatin capsules containing each 0.125 gm. (2 grs.) with an equal amount of olive oil, and are given one or two, two or three times a day. No unpleasant effects were noticed, while the psychic effect was apparent as well as was the antispasmodic.—*Therapeutic Progress.*

